# Pioglitazone alleviates lacrimal gland impairments induced by high-fat diet by suppressing M1 polarization

**DOI:** 10.1016/j.jlr.2024.100606

**Published:** 2024-07-26

**Authors:** Yu-Qing Chen, Yu-Chao Shao, Rui-Li Wei

**Affiliations:** Department of Ophthalmology, Changzheng Hospital of Naval Medicine University, Shanghai, China

**Keywords:** Macrophage polarization, high-fat die, extraorbital lacrimal gland, metabolic disorder, *PPAR-γ*

## Abstract

A high-fat diet (HFD) contributes to the pathogenesis of various inflammatory and metabolic diseases. Previous research confirms that under HFD conditions, the extraorbital lacrimal glands (ELGs) can be impaired, with significant infiltration of pro-inflammatory macrophages (Mps). However, the relationship between HFD and Mps polarization in the ELGs remains unexplored. We first identified and validated the differential expression of *PPAR-γ* in murine ELGs fed ND and HFD through RNA sequencing. Tear secretion was measured using the Schirmer test. Lipid droplet deposition within the ELGs was observed through Oil Red O staining and transmission electron microscopy. Mps phenotypes were determined through quantitative RT-PCR, immunofluorescence, and flow cytometric analysis. An in vitro high-fat culture system for Mps was established using palmitic acid (PA), with supernatants collected for co-culture with lacrimal gland acinar cells. Gene expression was determined through ELISA, immunofluorescence, immunohistochemistry, quantitative RT-PCR, and Western blot analysis. Pioglitazone reduced M1-predominant infiltration induced by HFD by increasing *PPAR-γ* levels in ELGs, thereby alleviating lipid deposition and enhancing tear secretion. In vitro tests indicated that *PPAR-γ* agonist shifted Mps from M1-predominant to M2-predominant phenotype in PA-induced Mps, reducing lipid synthesis in LGACs and promoting lipid catabolism, thus alleviating lipid metabolic disorders within ELGs. Conversely, the *PPAR-γ* antagonist induced opposite effects. In summary, the lacrimal gland is highly sensitive to high-fat and lipid metabolic disorders. Downregulation of *PPAR-γ* expression in ELGs induces Mps polarization toward predominantly M1 phenotype, leading to lipid metabolic disorder and inflammatory responses via the NF-κb/ERK/JNK/P38 pathway.

## Graphical abstract

**Figure:**
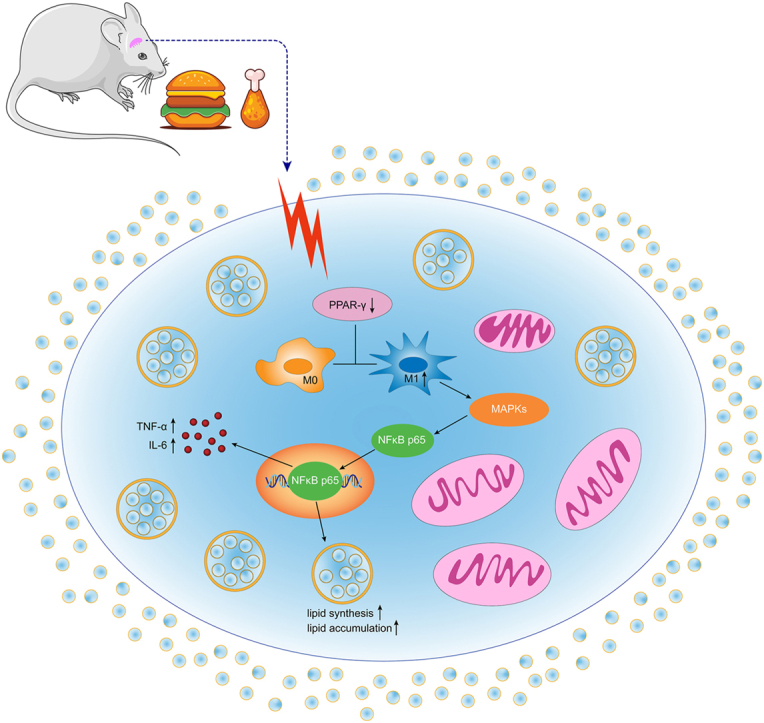

Global industrialization has induced changes to people’s lifestyles as witnessed by the high consumption of the Western diet (1, 2). Compared to normal dietary patterns, high-fat diet (HFD) significantly increases the risk of various diseases such as obesity (3), diabetes (3), and metabolic syndrome (4). Long-term consumption of HFD, along with nutritional excess and resultant obesity, is considered a trigger for chronic low-grade inflammation (LGI), previously referred to as 'X syndrome' (5), 'the deadly quartet' (6), and 'insulin resistance syndrome' (7). This phenomenon of persistent immune activation, in the absence of overt infections or autoimmune diseases, not only induces systemic chronic LGI through obesity but also manifests in local tissues, a condition known as meta-inflammation (8).

HFD significantly increases susceptibility of eye diseases (9, 10). Studies examining the potential pathogenesis of dry eye disease (DED) have revealed associations with elevated serum cholesterol levels. For example, a clinical study on a South Korean female population supported this association. (11). Another prospective cross-sectional study demonstrated a correlation between DED and obesity, suggesting that individuals with higher body fat levels are more likely to exhibit symptoms of DED (12). The ratio of two essential fatty acids, omega-6 to omega-3, in the daily diet, is approximately 1:1 (13). Recent clinical and epidemiological research have revealed a relationship between the dietary ratio of omega-6 to omega-3 and the risk of developing DED, especially among postmenopausal women (14, 15). Animal studies have demonstrated that an HFD and resultant obesity can induce structural changes and functional impairments in the cornea (16), conjunctiva (17), and meibomian glands (18), leading to a range of ocular damages similar to those found in DED and Meibomian gland dysfunction. While metabolic and lipid factors, including HFD, have garnered increased attention from researchers and clinicians, the impact of high-fat intake on normal ocular surface balance and diseases remains incompletely understood. There is an urgent need to further explore the effects of HFD on ocular health and develop effective treatment methods and intervention measures.

The lacrimal gland is an essential component of the ocular system and exerts its function by releasing aqueous tears (19, 20, 21). Despite the fact that the lacrimal gland plays a critical role, research on the impact of HFD on the lacrimal gland remains limited. Zou *et al.* (22) conducted high-throughput RNA sequencing of murine extra orbital lacrimal glands(ELGs) over a 24-h circadian cycle, revealing that an HFD can disrupt the circadian transcriptome profile and secretory rhythm of the ELGs. Additionally, He *et al.* (23) discovered that long-term excessive intake of an HFD induces pathophysiological changes in the murine ELGs, leading to a functional decline and often resulting in aqueous-deficient DED (24). Despite significant progress over the past two decades, DED research remains an evolving field with many questions to be explored, particularly the status of the lacrimal gland related to DED onset and development. Damages to the lacrimal gland typically involve extensive lymphocyte infiltration, acinar cell atrophy, and fibrotic changes. Moreover, any of these changes can disrupt the normal regulation of the lacrimal gland, ultimately triggering DED (25).

Since the relationship between obesity and inflammation has been established, researchers have gained numerous insights into how Mps reside in specific tissues and exhibit different activation states under the influence of metabolites. Mps serve as sentinels for immune activity and maintain homeostasis within the body, with their activation states changing in response to the environment, a process known as Mps polarization. M1 produce and secrete iNOS, TNF-α, and IL-1β. In contrast, M2, or alternatively activated Mps, highly express arginase (Arg)-1, produces and secrete anti-inflammatory factors like IL-10, CD206, and mannose receptor C type (Mrc) 2, playing roles in anti-inflammation and promoting tissue repair (26). In normal ELGs, Mps are resident cells of the innate immune system, with different phenotypes of Mps playing varying roles in regulating inflammatory responses of the ELGs and ocular surface. For instance, Lu *et al.* (27) found that infusion of human umbilical cord mesenchymal stem cells into a rabbit model with autoimmune dacryoadenitis suppressed M1 markers and increased M2 markers in the LGs.

The aforementioned previous findings support that the lacrimal gland can be used as an ideal model for investigating the relationship among an HFD, lipid metabolism, inflammatory responses, and Mps. Although significant research has been conducted on metabolism, HFD, and obesity, the impact of high-fat intake on the regulation of ocular surface disease has not been fully understood. This calls for further investigations into the effects of HFD on the ocular system and the development of effective treatment methods and interventions. Although evidence suggests that HFD can cause structural and pathological changes in the lacrimal gland (23), no study has shown a potential link between the lacrimal gland, HFD, and Mps polarization. Therefore, we investigated the potential mechanisms by which HFD affects the structure and functions of the lacrimal gland, and the role of Mps polarization in this process through RNA sequencing, in vivo animal models, and in vitro cell culture.

## Materials and Methods

### HFD mouse model and treatment protocols

Male C57BL/6 mice, aged 6 weeks, were purchased from the Naval Military Medical University's Experimental Animal Center in Shanghai, China. The mice were housed in a controlled SPF environment. All animal procedures were conducted in strict accordance with the principles of the Declaration of Helsinki and ARRIVE guidelines. The experimental protocol was approved by the Animal Ethical Committee of Navy Medical University and the Ethical Committee of Shanghai Changzheng Hospital (SHCZ12936). 60 eight-week-old mice were divided into two groups. One group received a standard chow diet (12% kcal fat) for 8 weeks (ND group, n = 20). The other group received a high-fat diet (HFD, 60% kcal fat, n = 40) for 8 weeks. Within the HFD group, half the mice (n = 20) received Pioglitazone (PIO, 100 mg/kg) orally every other day starting with HFD administration. The body weights of the mice were monitored and recorded weekly. After 8 weeks of dietary intervention, the mice were euthanized, and ELGs were extracted for subsequent experiments.

### RNA sequencing

ELGs were collected from three mice fed with both ND and HFD. Total RNA was extracted from the ELGs using the Trizol reagent. The concentration of the RNA was determined using a Qubit Fluorometer (Invitrogen) and an Agilent 2100 BioAnalyzer (Agilent Technologies). Sequencing libraries were prepared with the NEB Next Ultra RNA Library Prep Kit for Illumina, followed by library amplification via polymerase chain reaction (PCR). The prepared cDNA libraries were then sequenced on an Illumina Novaseq6000 platform at the Gene Denovo Biotechnology Co. Differentially expressed genes (DEGs) were selected based on Q-value <0.05 and a log2(Fold Change) > 1. Pathway enrichment of the DEGs was determined on the KEGG database.

### Measurement of aqueous tear secretion

After the dietary intervention, tear secretion was evaluated at 13:00 in a standard environment using the phenol red thread test (Jingming New Technology Development Co. Tianjin, China). Mice were anesthetized and the lower eyelid was gently pulled down, and the thread was carefully positioned on the palpebral conjunctiva, one-third of the way from the lateral canthus towards the lower eyelid, for 15 s. The distance covered by the red dye along the thread was calculated and presented in millimeters.

### Hematoxylin-eosin (HE) and Oil Red O staining

Murine ELGs were harvested and immediately fixed in 4% paraformaldehyde. Subsequently, they were embedded in the OCT compound, and cryosections with a thickness of 6 μm were prepared for further histological evaluation. Tissue sections were stained using Hematoxylin-eosin (G1004, Servicebio) or Oil Red O (G1016, Servicebio). The stained sections were then observed under a light microscope (Pannoramic 250 MIDI; 3DHISTECH Ltd).

### Transmission electron microscope

Murine ELGs were excised and rinsed in PBS. The tissues were then sectioned into 1 mm³ blocks and fixed in 2.5% glutaraldehyde at 4°C overnight. The ELG samples were prepared for Transmission electron microscope (TEM) following a previous protocol (23). Ultrastructural features of the ELGs were carefully examined and imaged using a TEM (Hitachi, HT7800, Japan).

### Immunofluorescence

6 μm thick ELG sections were fixed in 4% paraformaldehyde (PFA) at room temperature (RT) for 20 min and then washed in PBS before treatment with 0.2% Triton X-100 for 30 min to permeabilize the cells. Next, sections were then incubated with 2% BSA for 60 min to block non-specific binding, followed by overnight incubation at 4°C with primary antibodies against F4/80 (1:100, GB113373), iNOS (1:100, GB11119), and CD206 (GB113497), all purchased from Servicebio. The sections were washed three times with PBS, 10 min each time, and then incubated with fluorochrome-conjugated secondary antibodies (1:500, GB25404, Servicebio) for fluorescence visualization. The sections were counterstained with DAPI for nuclei labeling. The immunofluorescence staining results were analyzed using a fluorescence microscope.

### Immunohistochemistry

6 μm ELG sections fixed in formalin and embedded in paraffin on slides coated with xylene were dewaxed in xylene and then rehydrated in gradually decreasing concentrations of ethanol. The activity of endogenous peroxidases was blocked with 0.6% H_2_O_2_. To evaluate alterations in lymphocytes, the sections were treated with anti-CD45 (1:500, GB113886, Servicebio), and incubated at 37°C for 1 h. Subsequently, the sections were counterstained with Mayer's hematoxylin, mounted with glycerol, and observed under the microscope before being photographed.

### Plasma lipid profile analysis

Plasma samples were collected from mice centrifuged and stored at −80°C for subsequent analyses. The serum concentration of LDL, TG, CHO, and HDL was quantified utilizing an Automatic Biochemistry Analyzer (Shenzhen Rayto Life Science Co., Ltd).

### Cell culture and treatment

Bone marrow-derived macrophages (BMDMs) were isolated from the femurs of healthy C57BL/6 male mice and cultured in DMEM supplemented with 10% FBS and 40 ng/ml M-CSF (SRP3221, Sigma Aldrich) for 7 days. Cells that adhered with a purity exceeding 95% were utilized for subsequent experiments. To mimic a high-fat microenvironment, BMDMs were treated with Palmitic acid (PA, 0.5 mmol/L, P0500, Sigma Aldrich), while DMEM served as the normal control (NC). M1 polarization was induced by stimulation with 100 ng/ml lipopolysaccharide (LPS; L2630, Sigma Aldrich), and M2 polarization was achieved using 5 ng/ml interleukin-4 (IL-4; 214-14, Peprotech, USA) for a duration of 12 h. In addition, to explore the modulation of *PPAR-γ* activity, BMDMs were pre-treated with either the *PPAR-γ* agonist GW1929 (20 μmol/L, 370,695, Sigma Aldrich) or the *PPAR-γ* antagonist GW9662 (60 μmol/L, M6191, Sigma Aldrich) for 3 h before PA administration. Subsequently, supernatants were collected from these cell cultures, centrifuged, and filtered to remove impurities, to obtain a conditioned media (CM).

Following euthanasia, ELGs were extracted from the mice and rinsed in a sterile Petri dish. Primary lacrimal gland acinar cells (LGACs) were then isolated from these ELGs using established methods (28). After overnight incubation, the LGACs were incubated with PA (0.5 mmol/L), LPS (100 ng/ml), and IL-4 (5 ng/ml) for 24 h. The CM derived from BMDMs was utilized to establish a conditional co-culture system with LGACs. The following experimental groups were established: CM-NC, CM-LPS, CM-IL4, and CM-PA.

### ELISA

The concentrations of TNF-α and IL-6 in the supernatants obtained from the LGACs were determined using ELISA kits (MTA00B, and M6000B, R&D Systems), following the manufacturer's protocol.

### Flow cytometry

BMDMs were incubated with an anti-F4/80-FITC antibody (123,107, Biolegend) for 30 min at 4°C to achieve surface staining. Subsequent intracellular staining was conducted with anti-iNOS-APC antibody (696807, BioLegend). Fluorescence-activated cell sorting (FACS) data were collected and analyzed with FlowJo software.

### Total RNA isolation and real-time PCR

RNA was isolated from murine ELGs, BMDMs, and LGACs using TRIzol reagent (TaKaRa). The isolated RNA was then reverse-transcribed into cDNA with the PrimeScript RT Reagent Kit (TaKaRa). Real-time PCR assays were performed using 10 ng cDNA template in a 10 μl reaction mix containing specific primers and TB Green PCR Master Mix (TaKaRa). All PCR experiments were run in triplicate. The relative expression levels of target genes were determined using the 2^-ΔΔCt^ method. Primer sequences for murine genes were obtained from Shanghai Sangon Biotechnology Co., and are provided in Table 1.

**Table 1:** Primer Sequences for qRT-PCR

Gene	Forward Primer Sequence (5′-3′)	Reverse Primer Sequence (3′-5′)
*β-actin*	F: CATGTACGTTGCTATCCAGGC	R: CTCCTTAATGTCACGCACGAT
*PPAR-γ*	F: GGAAGACCACTCGCATTCCTT	R: GTAATCAGCAACCATTGGGTCA
*TNF-α*	F: CCTCTCTCTAATCAGCCCTCTG	R: GAGGACCTGGGAGTAGATGAG
*iNOS*	F: TTCAGTATCACAACCTCAGCAAG	R: TGGACCTGCAAGTTAAAATCCC
*IL-6*	F: ACTCACCTCTTCAGAACGAATTG	R: CCATCTTTGGAAGGTTCAGGTTG
*Arg-1*	F: TGGACAGACTAGGAATTGGCA	R: CCAGTCCGTCAACATCAAAACT
*Mrc2*	F: CCGAAACCGGCTATTCAACCT	R: CGGTCACACTCATACATGCCC
*CD206*	F: GGGTTGCTATCACTCTCTATGC	R: TTTCTTGTCTGTTGCCGTAGTT
*ACOX1*	F: AATCGGGACCCATAAGCCTTT	R: GGGAATACGATGGTTGTCCATTT
*CPT1α*	F: TCCAGTTGGCTTATCGTGGTG	R: TCCAGAGTCCGATTGATTTTTGC
*SREBP1C*	F: ACAGTGACTTCCCTGGCCTAT	R: GCATGGACGGGTACATCTTCAA
*FASN*	F: TTGGGTGCTGACTACAACCT	R: TGGATGATGTTGATGATGGA

*ACOX1*, acyl-CoA oxidase 1; *Arg1*, arginine-1; *CPT1α*, carnitine palmitoyltransferase 1α; *FASN*, fatty acid synthase; *IL*, interleukin; *iNOS*, inducible nitric oxide synthase; *Mrc2*, macrophage mannose receptor 2; *PPAR-γ*, peroxisome proliferator activated receptor gamma; *SREBP1C*, sterol-regulatory element binding protein 1C; *TNF-α*, tumor necrosis factor α.

### Western blot analysis

Murine ELGs and LGACs were lysed with RIPA lysis buffer (20–188, RMerck) containing a protease inhibitor to isolate total proteins. The proteins were transferred onto PVDF membranes, blocked with 5% BSA for 60 min and incubated overnight at 4°C with primary antibodies against PPAR-γ (1:1,000, AB45036, Abcam), SREBP1C(1:1,000, AB313881, Abcam), ACOX1(1:1,000, AB313881, Abcam), FASN (1:1,000, AB128870, Abcam), total and phosphorylated forms of ERK (1:1,000, 4695S and 4370S, respectively), JNK (1:1,000, 9252S and 4668S, respectively), P38 (1:1,000, 8690S and 4511S respectively), and NF-κB P65 (1:1,000, 8282S and 3033S, respectively) from CST. Subsequently, the samples were incubated at RT for 1 h with secondary anti-rabbit antibodies conjugated with horseradish peroxidase (1:5,000, 7074P2, Abcam). Following three washes with TBST, protein bands were detected using enhanced chemiluminescence (ECL,34,577, Thermo Fisher Scientific). The captured images were then analyzed by Image J software.

### Statistical analysis

Data were analyzed with GraphPad Prism software (version 9.5.0). Groups were compared with a two-tailed Student's *t* test and two-way analysis of variance (ANOVA). A *P*-value of < 0.05 was considered statistically significant. Data are presented as mean ± standard error of the mean (SEM).

## Results

### *PPAR-γ* is downregulated in the ELG of HFD mice, as evidenced by RNA sequences

**Fig. 1:**
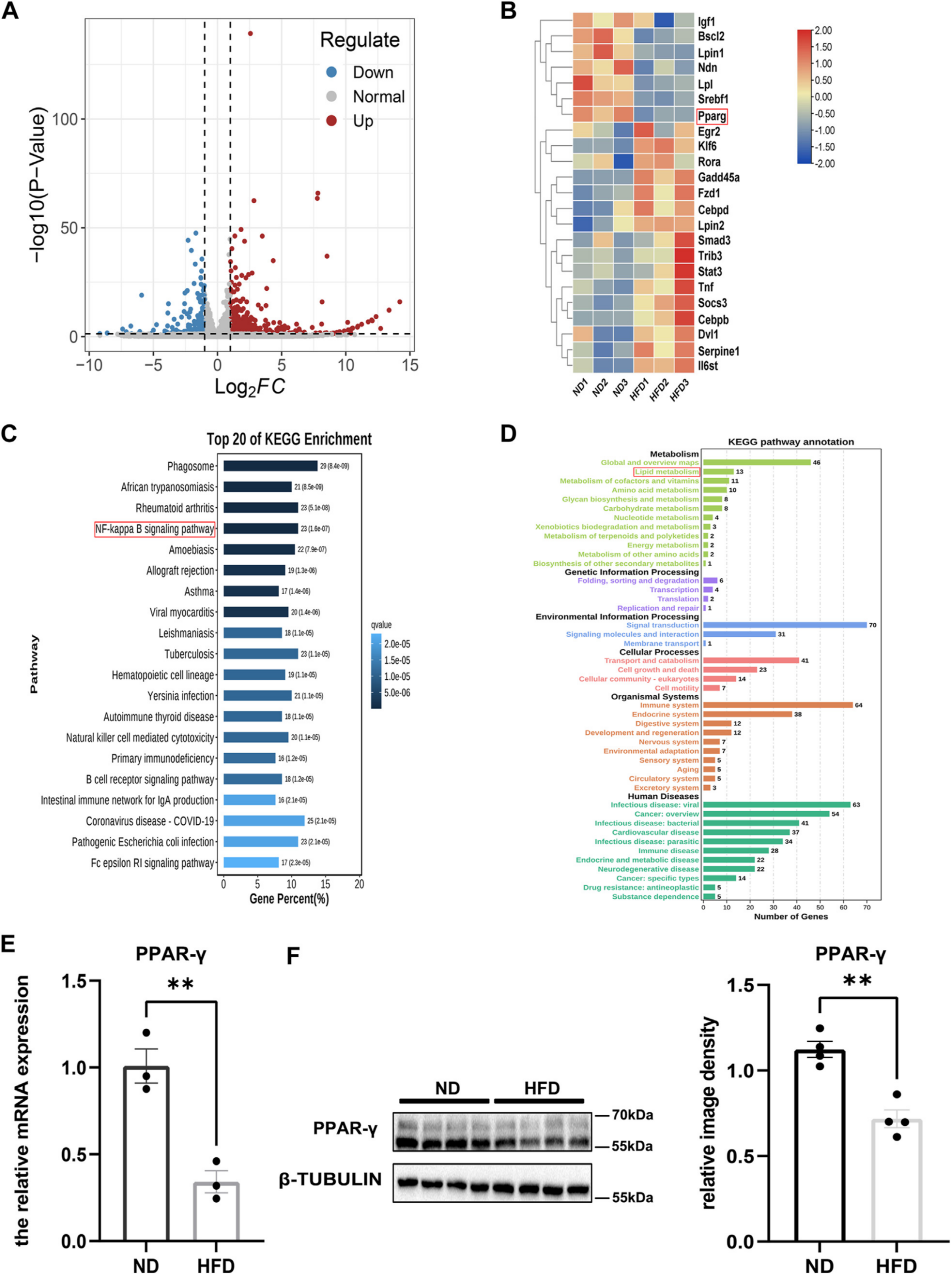
*PPAR-γ* was differentially expressed in the ELGs of HFD mice. A: the volcano plot displaying the differentially expressed genes (DEGs) between HFD and ND groups, with 284 upregulated genes and 114 downregulated genes. Upregulated genes are marked in red, whereas downregulated genes are marked in blue (n = 3). B: the heatmap displaying 23 lipid metabolism-related genes that were differentially expressed between the HFD and ND groups, with orange-red indicating elevated genes and blue indicating decreased genes. *PPAR-γ* is specifically highlighted within a red box. Data were normalized using the Z-score of log2- FPKM, with clustering based on Euclidean distance. C, D: KEGG pathway enrichment analysis of the DEGs. E: Real-time PCR analysis showing that *PPAR-γ* gene expression was down-regulated in the ELGs from the HFD group compared to the ND group (n = 3). F: Western blot analyses confirmed that PPAR-γ protein expression was lower in the ELGs of HFD mice (n = 4). ∗∗*P* < 0.01.

We investigated the gene expression profiles in ELG samples from mice fed an ND and an HFD, identifying 284 upregulated and 114 downregulated genes. The differential expression of these genes is illustrated in a volcano plot (Fig. 1A). Furthermore, we identified 23 differentially expressed lipid metabolism-related genes by overlapping the DEGs with a list of 130 lipid metabolism genes (supplemental Table S1). Notably, among these differentially expressed lipid metabolism-related genes, the down-regulation of *PPAR-γ*, a critical regulator of glycolipid metabolism and the immune response, was prominent (Fig. 1B). KEGG pathway enrichment analysis of the DEGs revealed significant associations, particularly with the NF-κB signaling pathway, ranking among the top 20 enriched pathways (Fig. 1C), and with metabolic pathways, especially those related to lipid metabolism (Fig. 1D). To validate the RNA-seq findings, we quantified *PPAR-γ* expression in ELGs from both HFD and ND groups using Real-time PCR and Western blotting. The mRNA levels of *PPAR-γ* in the HFD group were significantly lower than those in the ND group (Fig. 1E), a finding consistent with the protein levels of *PPAR-γ*, which were also significantly reduced in the HFD group (Fig .1F). These results corroborate the RNA-seq findings.

### Decreased tear secretion in HFD mice, alleviated by PIO

**Fig. 2:**
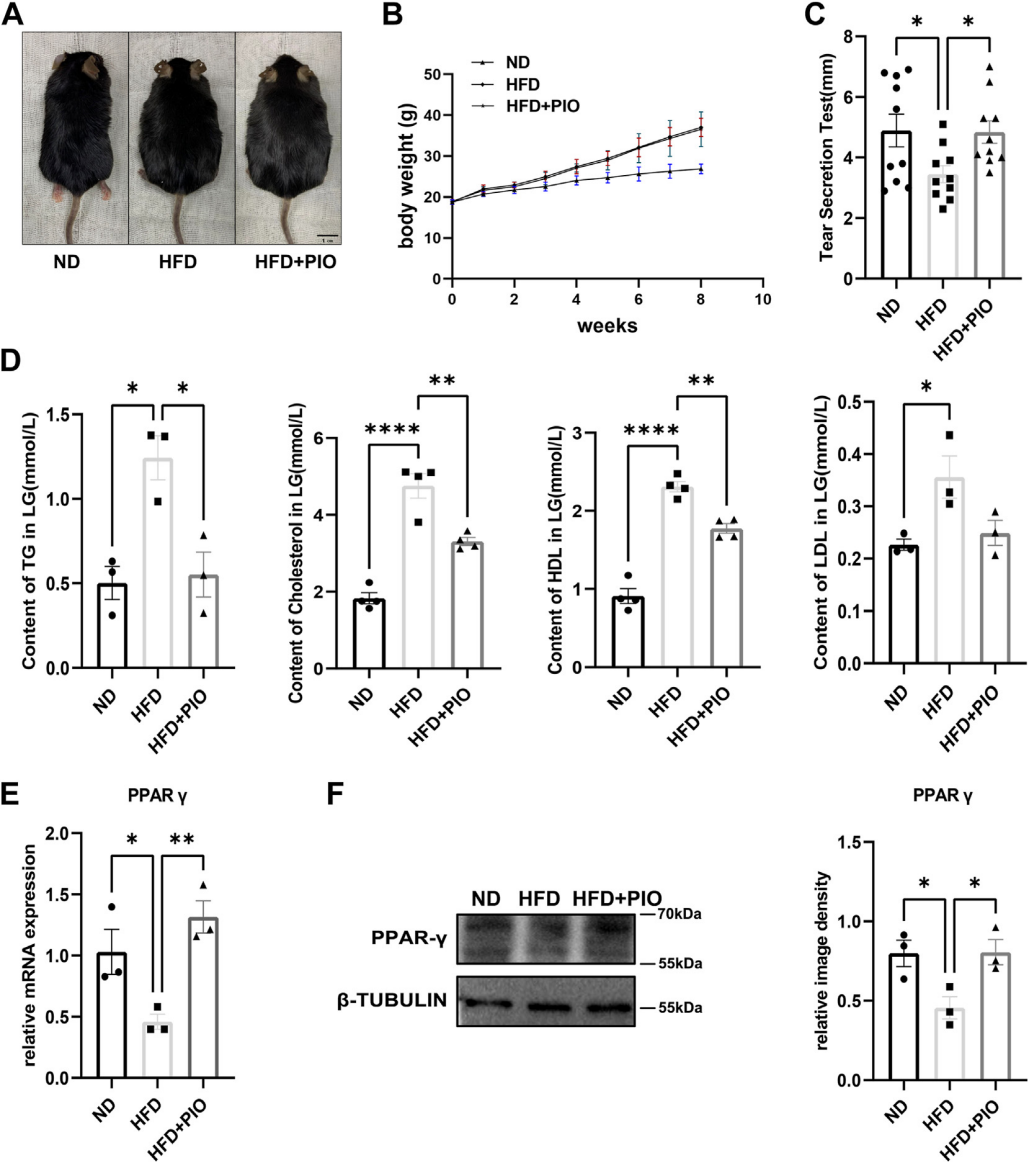
Tear secretion decrease in HFD mice, and PIO treatment alleviates tear secretion decrease induced by HFD. A: After 8 weeks, mice fed with HFD exhibited increased body size compared to those on a ND. Scale bars: 1 cm B: Body weight of mice (n = 20). C: Analysis of tear secretion using phenol red thread tests indicated decreased tear secretion in HFD mice, which was alleviated by PIO treatment (n = 10). D: Serum lipid levels were compared among mice fed with ND, HFD, and HFD supplemented with PIO (n = 3 or 4). E, F: Real-time PCR and Western blot analyses demonstrated changes in PPAR-γ expression levels in ELGs (n = 3). The data are presented as means ± SEM. ∗*P* < 0.05, ∗∗*P* < 0.01, ∗∗∗*P* < 0.001.

Eight weeks of HFD feeding resulted in increased body size (Fig. 2A) and weight (Fig. 2B) in mice compared to the ND group. PIO treatment did not significantly affect these measures. HFD decreased tear secretion (Fig. 2C), which partially improved with PIO administration, although not reaching normal levels. Furthermore, HFD elevated levels of TG, CHO, LDL, and HDL. PIO treatment significantly reduced the HFD-induced increase in TG, CHO, and HDL (Fig. 2D). Real-time PCR analysis revealed decreased expression of *PPAR-γ* in ELGs of the HFD group, while PIO treatment significantly increased *PPAR-γ* expression in these tissues (Fig. 2E). In line with these observations, Western Blot further confirmed that administration of PIO increased PPAR-γ expression in ELGs from mice fed with HFD (Fig. 2F). Taken together, these results suggest that supplementation with PIO may mitigate the decrease in PPAR-γ expression and tear secretion function caused by an HFD in murine ELGs.

### PIO treatment alleviates abnormal lipid metabolism in murine ELGs induced by HFD

**Fig. 3:**
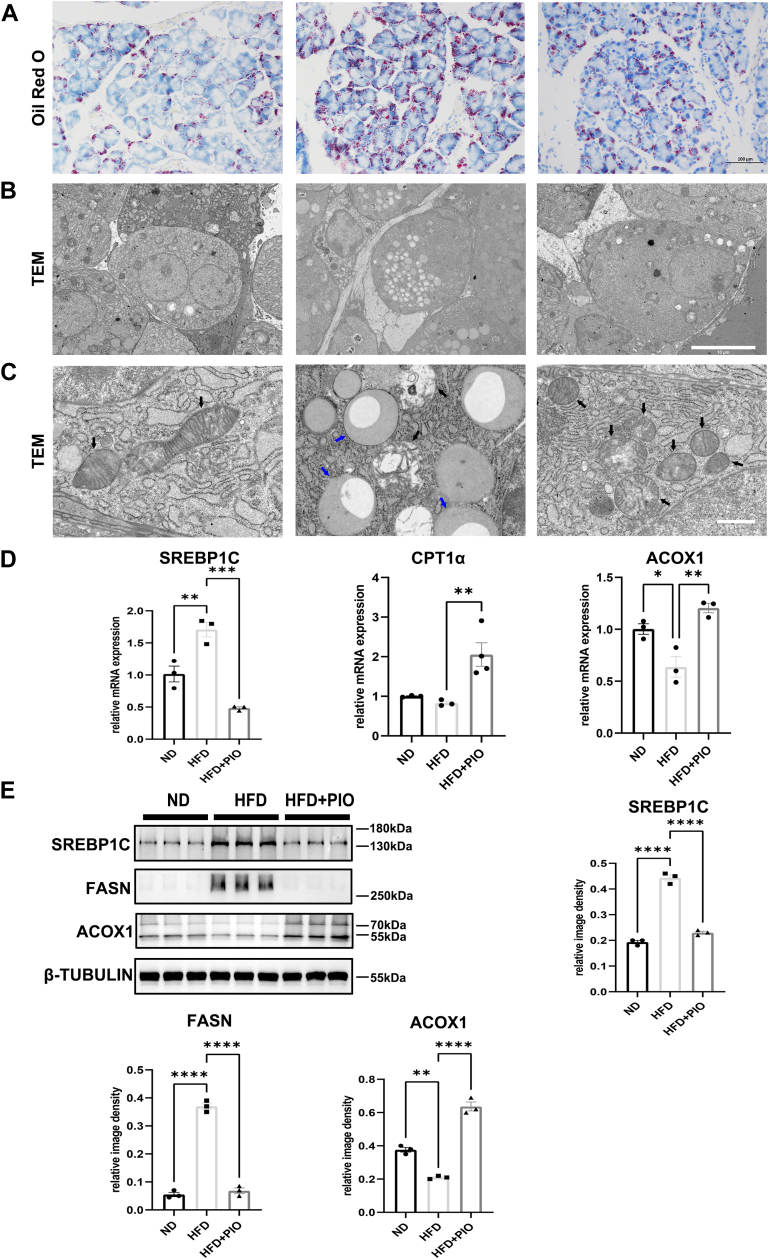
HFD induces lipid metabolism disorder in the ELGs. A: Lipid accumulation in the ELGs was examined by ORO staining (n = 3). B: Transmission electron microscopy (TEM) revealed the ultrastructure of acinar cells in the ELGs. C: TEM further detailed the ultrastructure of lipid droplets and mitochondria within the acinar cells of the ELGs. D: Real-time PCR analyzed the expression of lipid metabolism-related genes (n = 3 or 4). E: Western Blot analyzed the expression of lipid metabolism-related genes (n = 3). Data presented are the means ± SEM. ∗*P* < 0.05, ∗∗*P* < 0.01, ∗∗∗*P* < 0.001, ∗∗∗∗*P* < 0.0001. The blue arrows indicate lipid droplets and the black arrows indicate mitochondria. Scale bars: 100 μm (A); 10 μm (B); 1 μm (C).

Lipid accumulation within the ELGs was visualized using ORO staining, showing a significant increase in lipid droplets in HFD mice. However, PIO treatment reduced this lipid deposition, as evidenced by decreased ORO staining intensity (Fig. 3A). TEM analysis further confirmed a marked increase in lipid droplets within the cytoplasm of lacrimal acinar cells in HFD mice (Fig. 3B). Given the crucial role of mitochondrial enzyme activities in fatty acid oxidation, notable hypermegasoma, indicative of dysregulated mitochondrial metabolism, was observed in the ELGs of HFD mice (Fig. 3C), a sign of mitochondrial dysfunction as previously described (29). Notably, PIO treatment alleviated both the excessive lipid accumulation and mitochondrial swelling. RT-PCR analysis (Fig. 3D) further revealed that HFD increased the expression of *SREBP1C*, a gene involved in lipid synthesis, and decreased the expression of *ACOX1*, a gene involved in lipid breakdown. The expression of *CPT1α*, another lipid breakdown gene, remained unchanged. PIO treatment in the HFD group counteracted these effects, reducing SREBP1C expression and increasing the expression of both ACOX1 and CPT1α. Western blot analysis (Fig. 3E) confirmed these findings.

### PIO Mitigates inflammation in ELGs induced by an HFD

**Fig. 4:**
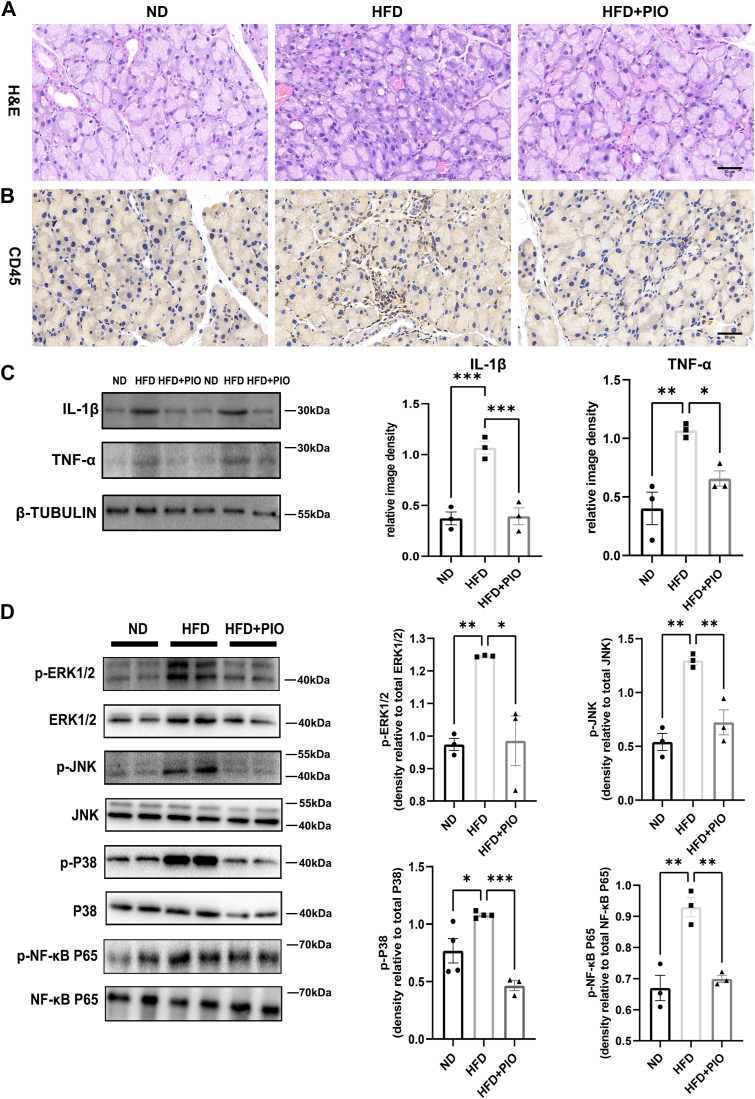
PIO Mitigates inflammation in ELGs induced by an HFD. A: H&E staining to determine the morphological alterations in the ELGs (Scale bar, 50 μm) (n = 3). B: IHC analysis of CD45 showing the presence of lymphocyte within the ELGs (Scale bar, 50 μm) (n = 3). C: Western Blot analyzed the expression of inflammatory proteins (n = 3). D: Western blot analysis indicating increased NF-κB p65, ERK, JNK, and p38 MAPK phosphorylation in mice ELGs following HFD treatment. Treatment with PIO alleviated the phosphorylation levels (n = 3 or 4). Data presented are the means ± SEM. ∗*P* < 0.05, ∗∗*P* < 0.01, ∗∗∗*P* < 0.001.

Murine ELGs from the HFD group displayed significant acinar atrophy and significantly greater lymphocytic infiltration compared to the ND group, as shown in Fig. 4A, B. This inflammatory response was significantly reduced in the PIO treatment group. Furthermore, HFD significantly increased the levels of inflammatory proteins TNF-α and IL-1β, while PIO treatment reduced their expression (Fig. 4C). Similar to previous observations, the NF-κB signaling pathway emerged as one of the top 20 pathways in the KEGG enrichment analysis. Subsequent investigations focused on the phosphorylation states of NF-κB p65, ERK, JNK, and p38 MAPK in the ELGs. Western blot analysis revealed elevated phosphorylation levels of NF-κB p65, ERK, JNK, and p38 MAPK in response to HFD, which was significantly reduced following PIO treatment, indicating a reduction in activated NF-κB p65/MAPK signaling pathway. Therefore, the downregulation of *PPAR-γ* may contribute to the exacerbation of HFD-induced inflammation in the ELGs (Fig. 4D).

### PIO modulates HFD-induced M1/M2 polarization shift in murine ELGs

**Fig. 5:**
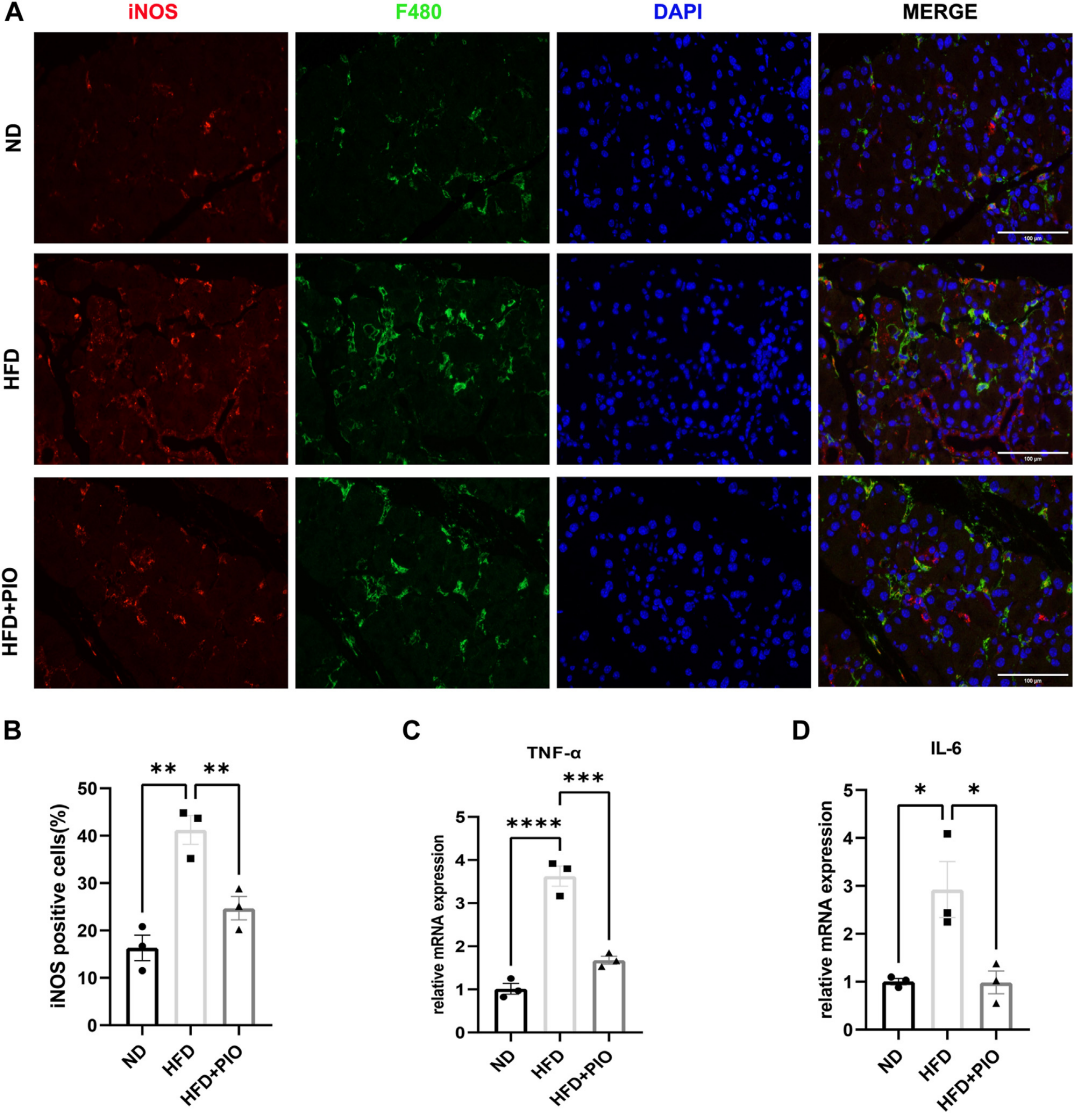
PIO modulates the HFD-induced M1/M2 polarization shifting in murine ELGs. A: IF staining for API (blue), F4/80 (green), and iNOS (red) illustrating differential expression in ELGs from ND, HFD, and HFD with PIO treatment (Scale bar, 100 μm). Histograms showing the proportion of Mps (F4/80+) positive for iNOS (n = 3). B: Histograms showing the proportion of Mps (F4/80+) positive for iNOS (n = 3). C, D: Real-time PCR assessed the expression levels of TNF-α and IL-6 in murine ELGs (n = 3). Data presented are the means ± SEM. ∗*P* < 0.05, ∗∗*P* < 0.01, ∗∗∗*P* < 0.001.

**Supplementary Figure 1:**
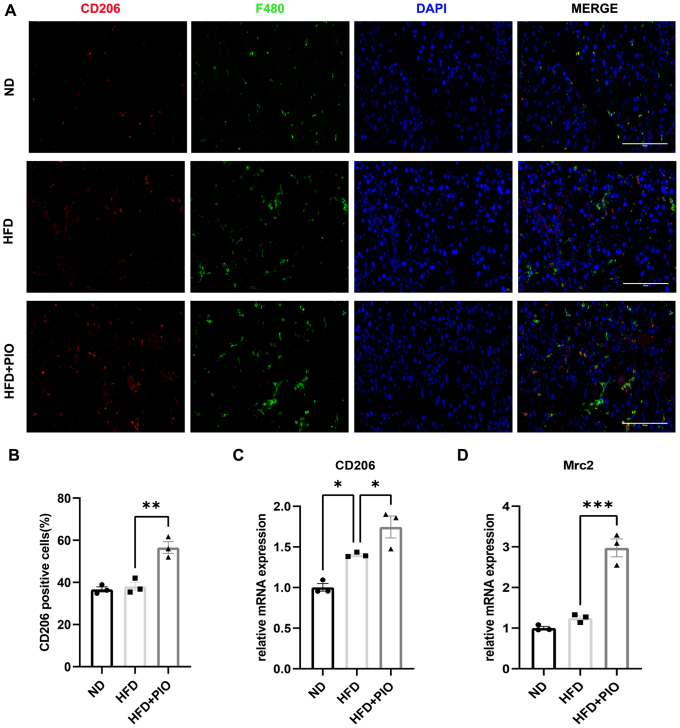
PIO modulates the HFD-induced M1/M2 polarization shifting in murine ELGs (A) IF staining for API (blue), F4/80 (green), and CD206 (red) illustrating differential expression in ELGs from ND, HFD, and HFD with PIO treatment (Scale bar, 100 μm) (n = 3). (B) Histograms showing the proportion of Mps (F4/80+) positive for CD206 (n = 3). (C, D) Real-time PCR assessed the expression levels of CD206, and Mrc2 in murine ELGs (n = 3). Data presented are the means ± SEM. ∗*P* < 0.05, ∗∗*P* < 0.01, ∗∗∗*P* < 0.001.

iNOS and CD206 are markers for M1 and M2 macrophages, respectively. HFD feeding significantly increased macrophage infiltration in murine ELGs. These infiltrated macrophages expressed higher levels of iNOS, a marker of M1 phenotype (Fig. 5A, B), but not CD206, a marker of M2 phenotype (supplemental Fig. S1A, B), compared to the ND group. Importantly, PIO treatment, which increased *PPAR-γ* expression in ELGs, significantly decreased M1 infiltration and a corresponding increase in M2 infiltration. Furthermore, real-time PCR analysis suggested a link between reduced *PPAR-γ* expression and the pro-inflammatory M1 macrophage infiltration observed in ELGs under HFD conditions (Fig. 5C, D and supplemental Fig. S1C, D).

### HFD promotes M1 predominant polarization in murine ELGs

**Fig. 6:**
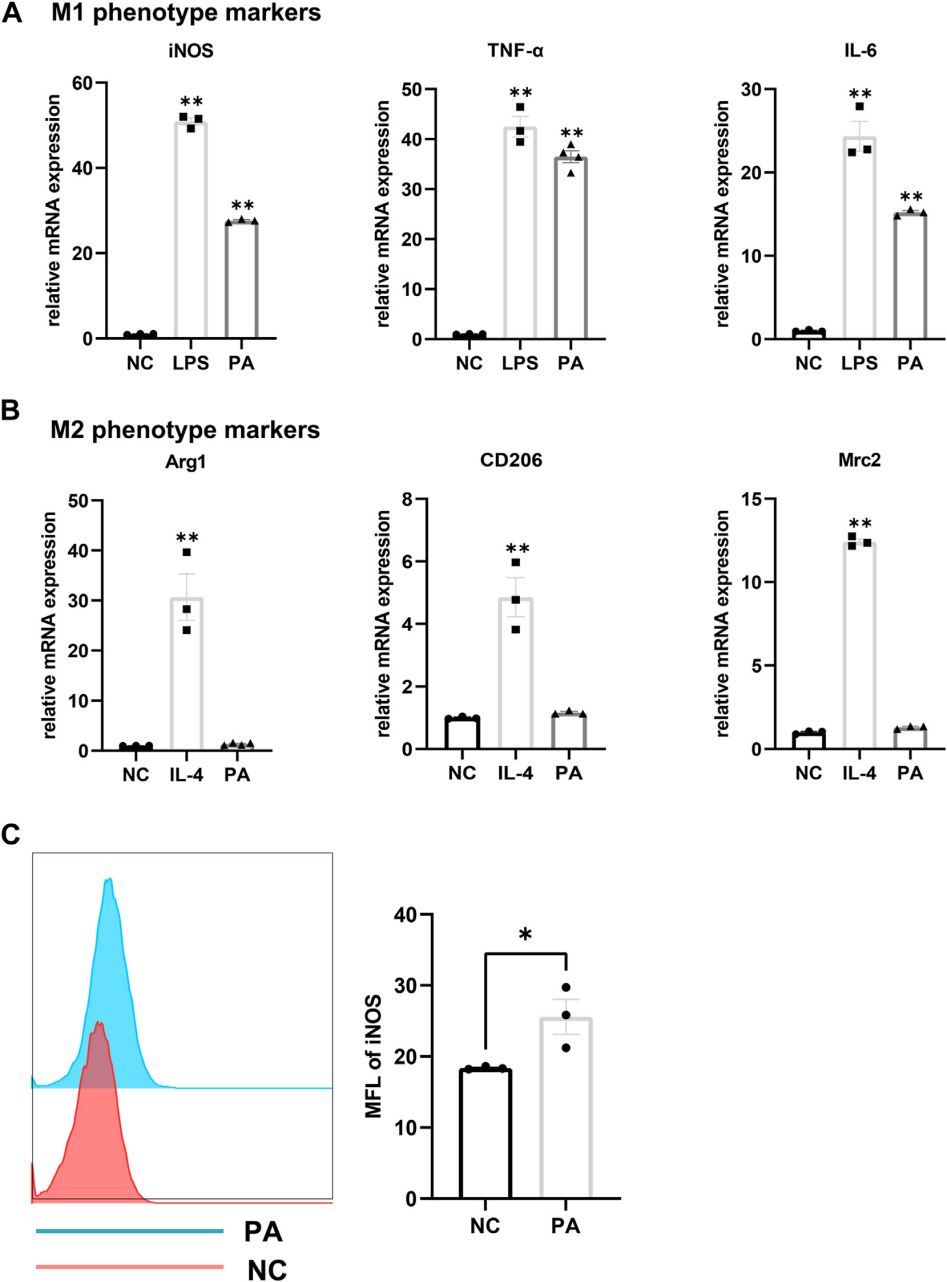
HFD induced a M1 predominant Mps polarization. A, B: BMDMs treated with PA at a concentration of 0.5 mmol/L constituted the PA group, while those cultured in DMEM served as the NC group. LPS at 100 ng/ml and IL-4 at 5 ng/ml served as positive controls to induce M1 and M2 polarization, respectively. Real-time PCR analysis of the expression levels of factors associated with M1/M2 polarization in treated BMDMs (n = 3). C: the flow cytometry analysis of iNOS expression in BMDMs (n = 3). Data presented are the means ± SEM. ∗*P* < 0.05, ∗∗*P* < 0.01. MFL, mean fluorescence intensity.

Considering the pivotal role of M1 polarization in inflammation and lipid metabolism within murine ELGs induced by an HFD, we investigated the potential of PA to influence Mps polarization in vitro. BMDMs were stimulated with PA, and the effects on Mps recruitment and phenotypic shift were examined. Notably, mRNA expression levels of M1 signature genes, including IL-6, TNF-α, and iNOS, significantly increased in response to PA stimulation (Fig. 6A). In contrast, the expression of CD206, Arg1, and Mrc2 (M2 macrophage-associated genes) was not changed following PA treatment (Fig. 6B). Flow cytometry further confirmed increased iNOS expression in PA-stimulated BMDMs relative to the NC group (Fig. 6C). Collectively, above results indicate the saturated fatty acid PA may drive Mps polarization towards a proinflammatory M1 predominant phenotype.

### Lipid-induced M1 polarization directly affects lipid metabolism and inflammation in LGACs by activating the NF-κB p65/MAPK signaling pathway

**Fig. 7:**
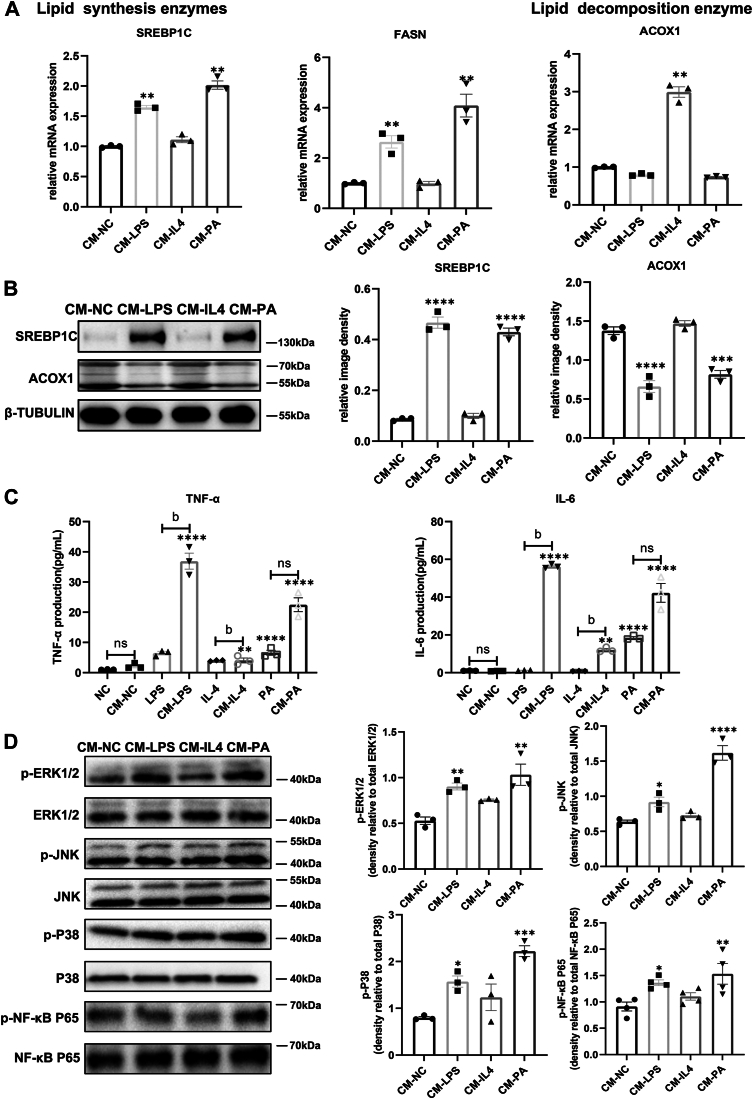
Lipid-induced M1 polarization has a direct effect on lipid metabolism and inflammation in LGACs BMDMs treated with PA at 0.5 mmol/L were assigned to the PA group, while those cultured in DMEM served as the NC group. LPS at 100 ng/ml and IL-4 at 5 ng/ml were employed as positive controls to induce polarization towards M1 or M2 phenotypes, respectively. Conditioned medium (CM) were collected from the cultured cells for incubation of LGACs for 24 h. A: Quantitative analysis of mRNA levels (n = 3). B: Protein levels of lipid metabolism-related genes in LGACs. PA treatment markedly increased the expression of SREBP1C and ACOX1(n = 3). C: Effect of PA-induced Mps polarization on PLACs inflammatory cytokine TNF-a and IL-6 secretion (n = 3). D: The expression of the NF-κB/MAPK signaling pathway genes in PLACs (n = 3). The data presented are the means ± SEM. ∗*P* < 0.05, ∗∗*P* < 0.01, ∗∗∗*P* < 0.001, ∗∗∗∗*P* < 0.0001 versus NC; a *P* < 0.05, b *P* < 0.01 relative to the designated two groups.

We confirmed that PA induces M1 polarization in Mps, leading to a significant increase in M1 markers, analogous to the effect observed with the positive control LPS. Next, we examined the effect of lipid-induced M1/M2 polarization on LGACs. CM from M1-dominant macrophages increased the expression of genes involved in lipid synthesis, such as SREBP1C and FASN, in LGACs. In contrast, CM from M2-dominant macrophages increased the expression of ACOX1, a gene involved in lipid breakdown, in LGACs (Fig. 7A, B). We further assessed the expression of pro-inflammatory cytokines, TNF-α and IL-6, in LGACs. The results were consistent with those observed with the positive control, LPS stimulation (Fig. 7C). Importantly, LGACs displayed activation of the NF-κB p65/MAPK pathway, as shown by increased levels of phosphorylated NF-κB p65, ERK, JNK, and p38 following treatment with LPS or PA (Fig. 7D).

### Regulation of PPAR-γ activity on lipid-induced M1/M2 shifting

**Fig. 8:**
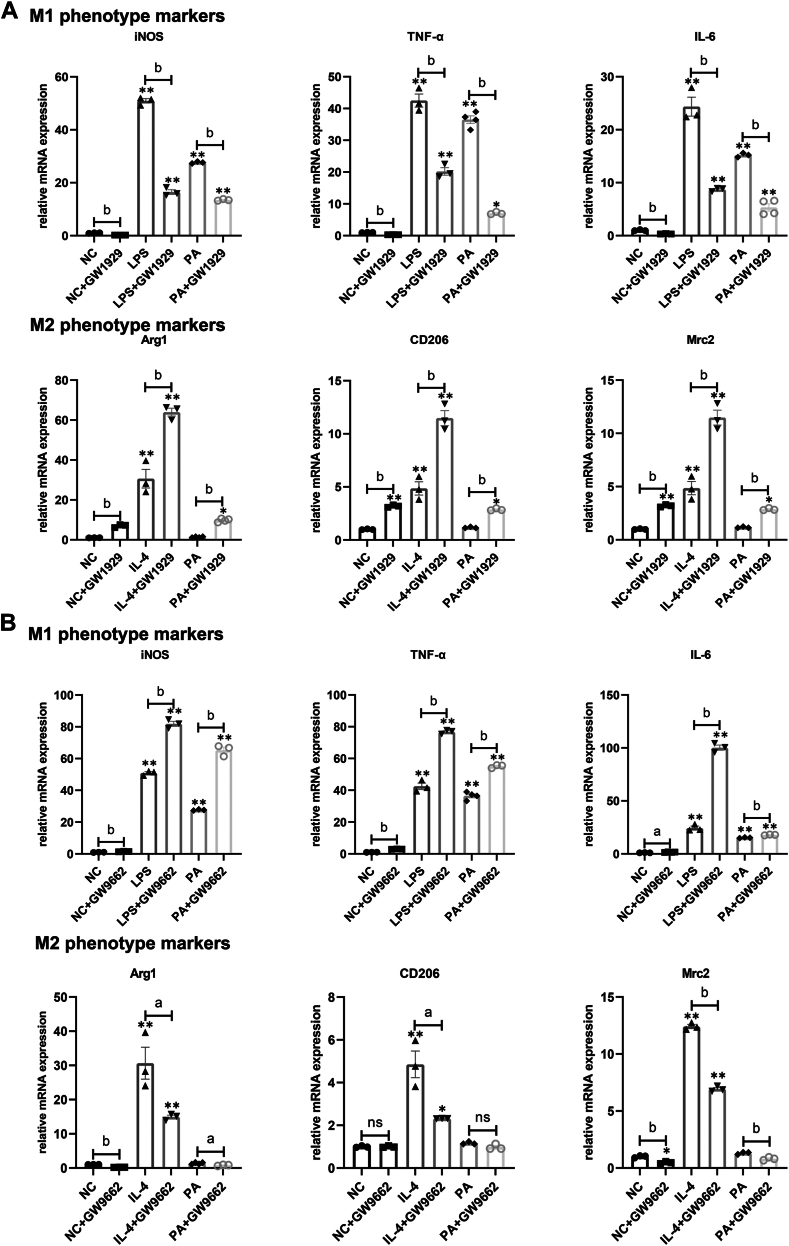
Impact of *PPAR-γ* modulation on lipid-induced M1/M2 shifting. Illustration of *PPAR-γ* agonist GW1929 (A) and *PPAR-γ* antagonist GW9662 (B) effects on the lipid-induced shift in Mps polarization from M1 to M2. BMDMs were pre-treated with either GW1929 or GW9662 for 3 h prior to exposure to PA at 0.5 mmol/L for an additional 12 h (n = 3). Data presented are the means ± SEM. ∗*P* < 0.05, ∗∗*P* < 0.01 versus NC; A *P* < 0.05, B *P* < 0.01 relative to the designated two groups.

Animal experiments have demonstrated that the down-regulation of *PPAR-γ* expression might contribute to the shift in M1/M2 polarization observed in ELGs induced by an HFD. To further elucidate the effect of *PPAR-γ* in PA-induced Mps, we treated BMDMs with either the *PPAR-γ* agonist GW1929, the *PPAR-γ* antagonist GW9662, or a combination of PA with either GW1929 or GW9662. As anticipated, GW1929 administration resulted in a reduction of M1 phenotype markers and upregulation of M2 phenotype markers in PA-treated BMDMs (Fig. 8A). Conversely, GW9662 treatment significantly decreased M2 phenotype markers while enhancing M1 phenotype markers in PA-treated BMDMs (Fig. 8B). These findings suggest that activation of *PPAR-γ* can modulate lipid-induced Mps polarization from the M1 to M2 phenotype, while inhibition of *PPAR-γ* exerts the opposite effect.

### *PPAR-γ* regulation attenuates lipid synthesis in LGAC by modulating lipid-induced M1/M2 shifting

**Fig. 9:**
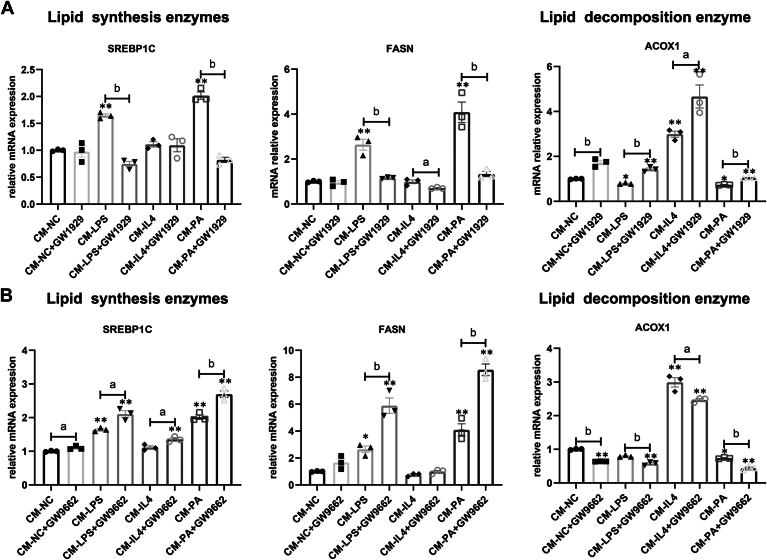
*PPAR-γ* attenuates the lipid synthesis in PLAC via regulating lipid induced M1/M2 shifting. BMDMs were pre-treated with GW1929 or GW9662 before exposure to PA for 12 h. The resulting CM were used for LGACs incubation. A: analysis of lipid metabolism-related gene expression in LGACs treated with CM from BMDMs pre-treated with GW1929 (n = 3). B: analysis of lipid metabolism-related gene expression in LGACs treated with CM from BMDMs pre-treated with the *PPAR-γ* antagonist GW9662 (n = 3). ∗*P* < 0.05, ∗∗*P* < 0.01 versus NC; a *P* < 0.05, b *P* < 0.01 relative to the designated two groups.

Given the ability of *PPAR-γ* activity to influence lipid-induced shifts in Mps polarization, we investigated the downstream effects of *PPAR-γ*-mediated Mps polarization on lipid metabolism in LGACs. LGACs were treated with CM derived from BMDMs previously exposed to LPS, IL-4, PA, GW1929, or GW9662, either individually or in combination. CM from Mps pre-treated with PA or LPS significantly elevated the mRNA levels of lipid synthesis-associated genes, including SREBP1C and FASN, in LGACs. However, these increases were significantly attenuated when LGACs were exposed to CM from Mps pre-treated with the *PPAR-γ* agonist GW1929 (Fig. 9A). Conversely, CM from Mps pre-treated with IL-4 enhanced the expression of ACOX1 in LGACs. As expected, the expression levels of ACOX1 were significantly reduced when LGACs were treated with CM from Mps pre-treated with the *PPAR-γ* antagonist GW9662 (Fig. 9B). In conclusion, these findings highlight the critical role of macrophage polarization shifts in regulating lipid metabolism within LGACs. By modulating macrophage polarization through *PPAR-γ* activity, it may be possible to significantly influence lipid metabolism pathways in these cells.

## Discussion

Currently, research on the effects of an HFD on lacrimal gland function and its underlying mechanisms is relatively limited. Although some studies have suggested that an HFD can induce structural and pathological changes in the lacrimal glands, none has demonstrated a potential link between lacrimal glands, HFD, and Mps polarization. In our study, we found that HFD-induced downregulation of *PPAR-γ* expression in the ELGs promoted pro-inflammatory M1 polarization, and lipid synthesis and inhibited lipid decomposition, which may be an important mechanism of HFD-induced inflammatory infiltration and lipid accumulation in the ELGs. The NF-κB/ERK/JNK/P38 signaling pathway may mediate these pathological changes induced by HFD. In vivo experiments showed that HFD induced lipid metabolism dysfunction in the murine ELGs, causing excessive deposition of lipids and inflammatory cell infiltration, which ultimately impairs the secretion of tears. Pioglitazone treatment could alleviate HFD-induced pathological changes in the murine ELGs. Collectively, our results reveal that decreasing *PPAR-γ* by HFD triggers lacrimal gland dysfunction via promoting pro-inflammatory M1 macrophage polarization.

Using RNA sequencing, we first detected differential expression of the *PPAR-γ* gene within the ELGs of mice fed to an HFD. To validate our findings, we assessed *PPAR-γ* expression in ELGs from HFD mice using real-time PCR and Western blot techniques. *PPAR-γ* is a critical nuclear receptor transcription factor that regulates insulin sensitivity, lipid metabolism, and blood glucose balance (30, 31). Increasing evidence suggests that *PPAR-γ* also regulates the response of immunocytes, including Mps (32, 33). The absence of *PPAR-γ* expression in Mps of obese individuals can lead to increased inflammation and insulin resistance (34). Additionally, *PPARs* play a significant regulatory role in Mps polarization (35, 36). Thiazolidinedione, a class *PPAR-γ* agonist, stands among the most effective insulin-sensitizing drugs currently known (37, 38). Considering our findings from the initial part of the study, where *PPAR-γ* gene expression was downregulated in the murine ELGs on an HFD, we administered *PPAR-γ* agonist PIO via gavage to HFD mice to activate *PPAR-γ* and modulate the effects of the HFD on the ELGs. As expected, PIO gavage significantly alleviated inflammatory infiltration, and lipid droplet accumulation in the ELGs in HFD mice, ultimately improving tear secretion. These results suggest that PIO may be beneficial in treating ELG damage and dry eye resulting from HFD. Additionally, fenofibrate has been explored in studies on ocular lesions in mice induced by HFD. He *et al.* (23) found that *PPAR-alpha* expression in the lacrimal glands began to decrease in the fourth month of HFD feeding, and fenofibrate treatment could reduce inflammation and lipid droplet deposition in the lacrimal glands. We hypothesize that apart from mitigating dyslipidemia induced by HFD and thus alleviating damage caused by systemic dyslipidemia to local ELGs, pioglitazone can activate *PPAR-γ* receptors within the ELGs themselves. This activation could initiate downstream signaling pathways, leading to improved lipid metabolism and resolution of functional disorders in the ELGs.

*PPARs* play a critical role in regulating macrophage polarization (33, 34). Activated macrophages express high levels of *PPAR-γ* (37). Studies have shown that blocking the *PPAR-γ* pathway reduces pro-inflammatory cytokine production by macrophages (38). Macrophages are implicated in the pathogenesis of ocular diseases, and their interactions with other immune cells are an area of ongoing research (39, 40). Oxygen-induced retinopathy (OIR) in mice serves as a common model for studying retinal neovascularization (RNV) (41). Studies indicate that both M1 and M2 types contribute to RNV formation at different stages, suggesting that modulating macrophage polarization can influence RNV generation (42, 43). In OIR mice, pigment epithelium-derived factor inhibits Mps polarization, suppressing RNV formation via activation of the MAPK and Notch1 pathways, through the inhibition of triglyceride lipase (42). In our experiments, we observed downregulation of the *PPAR-γ* gene in the ELGs of mice after 8 weeks on an HFD. The onset and progression of DED represent a continuous and complex pathophysiological process, where the mechanisms among various cells involved are tightly interconnected (44). He *et al.* (23) demonstrated that in murine ELGs fed an HFD, the F4/80+ Mps increases. This increase is closely associated with elevated levels of M1-type inflammatory cytokines IL-1β and TNF-α, as well as the degree of inflammation in the lacrimal gland, directly impacting the pathological changes. In our animal study, we found that an HFD significantly increases the M1 polarization markers in the ELGs, with a slight increase in the M2 polarization marker CD206, suggesting a partial effect on M2 polarization by the HFD. However, it predominantly induces a mixed phenotype of Mps with an M1 predominant polarization in the murine ELGs, leading to an imbalance in the ratio of M1/M2 type. Our study indicates that in mice fed an HFD, Mps in the ELGs are predominantly M1 polarized, and treatment with PIO significantly reduces M1-type, suggesting that the low expression of *PPAR-γ* induced by HFD may contribute to the formation of pro-inflammatory M1-type in the ELGs and a series of pathological changes. Additionally, in the earlier part of our study, through RNA sequencing, we performed KEGG enrichment analysis on DEGs, which indicated differential expression of the NF-κB signaling pathway in the ELGs of HFD and ND mice. Western blot analysis revealed that HFD downregulates PPAR-γ expression, potentially activating the NF-κB/ERK/JNK/P38 signaling pathways in ELGs. This suggests that *PPAR-γ* may play a role in regulating both lipid metabolism and inflammatory activity within these tissues. Consequently, HFD may lead to increased lipid accumulation and inflammation. However, further research is needed to elucidate the specific mechanisms involved.

We observed that an HFD induces dysfunction in lipid metabolism in murine ELGs, leading to abnormal lipid deposition, inflammatory cell infiltration, mitochondrial damage, and ultimately impairing ELG production and secretion functions. Dysregulation of lipid metabolism and inflammatory responses are critical influencing factors for ELG functions (23, 45). We further investigated the effect of a high-fat microenvironment, simulated by fatty acids in vitro, on BMDM polarization (46). PA, a saturated fatty acid, commonly induces polarization of liver Kupffer cells in models of alcoholic fatty liver disease. Initially, we stimulated primary Mps with PA in vitro and observed that PA promotes a dominant M1-type polarization. Next, we investigated the effect of PA-induced changes in macrophages on LGACs. CM-PA treatment resulted in decreased lipid catabolism and increased lipid synthesis in LGACs. This finding aligns with our in vivo observations in mice, where HFD feeding led to increased expression of SREBP1C, a gene involved in lipid synthesis, and decreased expression of ACOX1, a gene involved in lipid breakdown, in murine ELGs. Therefore, we propose that PA, by inducing M1-type polarization, upregulates lipid synthesis and downregulates lipid catabolism, promoting lipid accumulation in ELGs and mediating chronic damage. Studies have reported significantly increased expression of SREBP1C and its downstream target genes FASN and ACC1 in patients with NAFLD (47). Additionally, we observed elevated levels of inflammatory response factors in PA-treated LGACs, and significantly increased phosphorylation levels of NF-κB and its downstream proteins ERK/JNK/P38. Given the critical role of NF-κB signaling in metabolic regulation and inflammatory responses (48), we suggest that PA promotes lipid deposition by fostering M1-type polarization and activating the NF-κB/ERK/JNK/P38 signaling pathway, inhibiting fatty acid oxidation in LGACs, increasing lipid synthesis, and mediating inflammatory responses in murine ELGs.

We further investigated how variations in *PPAR-γ* expression levels in Mps influence their polarization state and subsequently affect lipid synthesis and catabolism processes in LGACs. Results revealed that the *PPAR-γ* agonist GW1929 significantly inhibited the increase in lipid synthesis in LGACs induced by M1-type, while the *PPAR-γ* antagonist GW9662 inhibited the increase in lipid catabolism induced by M2-type. These findings suggest that *PPAR-γ* agonists can reduce the stimulation of lipid synthesis metabolism in LGACs induced by PA by promoting the transition of Mps from M1 to M2 type. Conversely, *PPAR-γ* antagonists reduce lipid catabolism in PLAC by inhibiting M2-type polarization. In summary, increasing *PPAR-γ* expression can induce Mps polarization toward the anti-inflammatory M2 type, suppressing the increase in lipid synthesis and promoting lipid catabolism, thereby alleviating lipid metabolic abnormalities in the lacrimal gland. Conversely, downregulating *PPAR-γ* expression in Mps produces the opposite effect. As observed in both animal experiments and cell experiments, HFD-induced downregulation of *PPAR-γ* expression in the ELGs induces pro-inflammatory M1 polarization, promoting lipid synthesis and inhibiting lipid catabolism, which may be a key factor in HFD-induced inflammatory infiltration and lipid accumulation in the ELGs. The NF-κB/ERK/JNK/P38 signaling pathway may mediate these pathological changes induced by HFD. Activation of this pathway, coupled with lipid droplet accumulation and inflammatory cell infiltration, alters the ELG microenvironment, ultimately leading to reduced tear secretion. Our study highlighted the significance of *PPAR-γ* in modulating Mps polarization and its importance in lipid metabolism regulation, providing a critical theoretical basis for further biomedical research and potential clinical applications. By modulating *PPAR-γ* expression and Mps polarization, we may more effectively manage ocular and systemic diseases related to lipid metabolism abnormalities.

This study has several limitations. Firstly, although PIO acts as a *PPAR-γ* agonist, and we used it to intervene in mice on an HFD, verifying its ability to upregulate *PPAR-γ* transcriptional and translational expressions in murine ELGs, its role in alleviating ELG lesions induced by HFD may be influenced by other pathways due to its function as an important insulin-sensitizing medication and regulator of bodily glucose and lipid metabolism. Secondly, future studies could incorporate systemic metabolic outcomes, and compare them with changes in metabolism and function within the lacrimal gland.

**Fig. 10:**
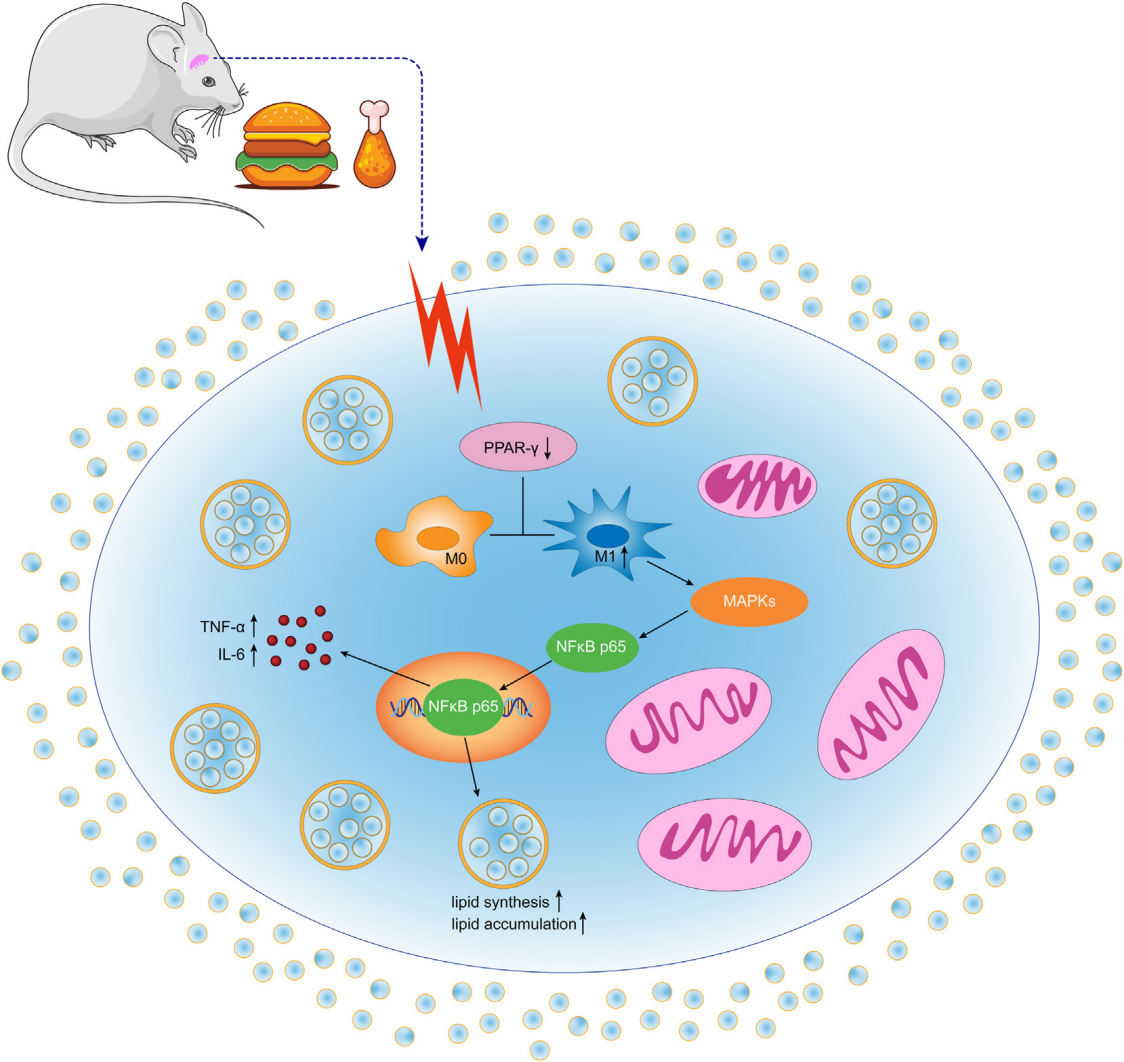
A proposed model of the PPAR-γ/M1 macrophage polarizations induced by high-fat diet.

In summary, the lacrimal gland displays high sensitivity to high fats and lipid metabolism disorders. Downregulation of *PPAR-γ* expression in the lacrimal gland induces a dominant M1-type polarization. This, in turn, may activate the NF-κb/ERK/JNK/P38 pathway, leading to lipid metabolism disorders and inflammatory responses (Fig. 10). This mechanism may contribute to the pathological changes induced in the lacrimal gland by a HFD. These findings indicate new therapeutic avenues for preventing and treating lacrimal gland or ocular lesions associated with HFD. The use of *PPAR-γ* agonists and the inhibition of specific pathway molecules could be valuable strategies in this regard.

## Data availability

Data will be made available on request.

## Ethics approval and consent to participate

The study was conducted in accordance with the Declaration of Helsinki and approved by the Committee of Ethics of Shanghai Changzheng Hospital.

## Supplemental data

This article contains supplemental data.

## Conflict of interest

The authors declare that they have no conflicts of interest with the contents of this article.
